# The Effect of Granulocyte Colony Stimulating Factor Administration on Preterm Infant with Neutropenia and Clinical Sepsis: A Randomized Clinical Trial

**Published:** 2013-04-22

**Authors:** L Borjianyazdi, M Froomandi, M Noori Shadkam, A Hashemi, R Fallah

**Affiliations:** 1 Assistant Professor of Neonatology, Shahid Sadoughi University of Medical Sciences and Health Services,Yazd, Iran.; 2 Pediatric Resident, Shahid Sadoughi University of Medical Sciences and Health Services, Yazd, Iran.; 3 Associate Professor of Neonatology, Shahid Sadooghi Medical University , Yazd, Iran.; 4 Department of Pediatrics, Hematology, Oncology and Genetics Research Center, Shahid Sadoughi University of Medical Sciences and Health Services,Yazd, Iran.; 5 Aassociate Professor of Pediatric Neurology, Shahid Sadoughi University of Medical Sciences and Health Services, Yazd, Iran.

**Keywords:** Granulocyte Colony-Stimulating Factor, Infant, Premature, Neutropenia, Sepsis

## Abstract

**Background:**

This study was conducted to evaluate the clinical effect of Granulocyte Colony Stimulating Factor (GCSF) on prognosis of neonatal sepsis.

**Materials and Methods:**

Present study is a double- blinded randomized clinical trial, conducted on 46 preterm infants with neutropenia (Absolute Neutrophil Count (ANC) ≤ 5000 / μL) and clinical sepsis. Infants were randomly allocated into two groups. In the first group (treatment group), infants were treated with GCSF for up to 5 consecutive days with 10 μg/ kg in addition to standard treatment protocols, and in other group, infants received normal saline as the placebo. Each infant was monitored for 14 days. Primary outcome was mortality during 14 days after entering the study, and secondary outcome was the incidence of positive blood culture, weight gain on the fourteenth day, the duration of hospitalization and medication side effects.

**Results:**

In the treated group, only one death was observed (P-value=1.00). However, no positive results for cultures were reported.

Only one case in the treatment group and 3 patients in the control group showed feeding intolerance and needed respiratory support (P-value= 0.608). Length of hospitalization was 25 ± 6 days for the treatment group and 30 ± 7 days for the control group which was statistically significant (P-value=0.042).

**Conclusion:**

The results of this study demonstrated that GCSF could reduce the hospital stay, but no significant effect was observed on mortality rate, respiratory or feeding status.

## Introduction

Neonatal Mortality Rate (NMR) is considered as an indicator of the health status of a population. According to UNICEF reports in 2010, neonatal mortality rate was 14 deaths per each 1,000 births in Iran, while this was 4 per 1,000 in the United States (1). Prematurity (birth before the end of 37th weeks of gestation) is considered as the main cause of 60 to 80% of mortality in infants without congenital anomalies (2). Despite numerous advances in the identification of new antibiotics and improved neonatal intensive care, neonatal sepsis remained as an important cause of mortality and morbidity in infants. Incidence of neonatal sepsis is reported to be1 to 5 cases per each 1,000 live births. According to reports, case fatality rate of neonatal sepsis in early (during the first 7 days of birth) and late sepsis (from day 7 to day 28) are 5 to 20% and 5%, respectively (3). Prematurity is the most important factor that increases the risk of infection in neonates and finally leads to death. The overall incidence of neonatal sepsis is 3 to 10 times greater in premature infants compared to infants with normal weight (4, 5).According to report from the National Institute of Child Health and Human Development (NICHD) Neonatal Research Network, infants with Extremely Low Birth Weight (ELBW)who have experienced a neonatal infection in the neonatal period , compared with infants who have not sepsis attack at follow up, are at higher risk of adverse neurodevelopmental outcome and poor growth (6). Therefore, diagnosis and appropriate treatment of sepsis in this group is essential to reduce mortality rate and improve the quality of their life.

Premature infants have an increased susceptibility to infection (7). In the presence of severe neutropenia (ANC ≤ 500/μL), mortality rate followed by sepsis increases by more than 50% (8).The average range of ANC, which is a standard criterion for definition of neutropenia, in neonatal period, is 11,000 cells/µL, but it varies between 6,000 and 26,000cells /µL. Therefore, according to the reported values of absolute neutrophil counts and based on similar studies (4), we described neutropenia as ANC of less than 5,000 cells /µL.

In the past two decades, researchers have tried to design methods to improve the immune system of premature infants with neutropenia by immunotherapy (use of subcutaneous GCSF or intravenous immunoglobulin (IVIG)) (9, 10, and 11). Based on previous studies, GCSF administration was associated with increased neutrophil count, and improved bactericidal function of neutrophils (12, 13, 14). In this study, we tried to evaluate the clinical efficacy of GCSF on prognosis of premature infants with neutropenia.

## Materials and Methods

This study was a double-blind, randomized , clinical trial conducted on preterm infants who were admitted to the Neonatal Intensive Care Unit (NICU) of Shahid Sadoughi Hospital (Yazd-Iran) from October 2012 to March 2013 .

Eligible participants were infants with:

- gestational age between 30 - 37 weeks
- chronological age less than 10 days
- clinical manifestation of clinical sepsis
- neutropenia (ANC ≤ 5000 / μL)

Sample size was assessed on 23 infant per group, based on Z formula and confidence interval of 95% with 80% power and type one error of 5%. Clinical sepsis is defined by any one of the followings in the absence of an alternative explanation: either fever (central temperature of > 38˚C) or two or more of the following :poor perfusion; persisting metabolic acidosis (base excess > −8 mmol/l ); increasing ventilation or supplemental oxygen to maintain adequate gas exchange over a minimum of four hours; > 25% reduction in platelet count from baseline or lower limit of normal; persisting glucose imbalance (<40 />180 mg/dl for four hours in spite of corrective measures); abdominal signs (abdominal distension, blood in stool, or bilious aspirates) (15). Infants with major anomalies, total leukocyte count ≥ 20.000/ mm³ (15), intra ventricular hemorrhage (grade ≥ 3), thrombocytopenia (Plt≤150,000/ μL), anemia (Hb≤10g/dL), metabolic acidosis (pH≤7.2) or sever asphyxia (grade 2 or 3) were excluded from study. After approval of study by the research ethics committee and informed written parental consent, infants who had the inclusion criteria, were enrolled in this study. First, laboratory tests of CBC (Diff), ANC, CRP and blood and urine culture were requested, and BD BACTEC Peds Plus/F culture vials (containing resin) were used for blood culture (in the laboratory of Sina). In order to eliminates any possibility of bias, infants were randomly (using table of random numbers) divided into two groups, and GCSF or placebo (normal saline solution) were identified to the investigators by only a code. For infants in case group, 10μg/kg/day of GCSF (Darou Pouyesh Co) was injected subcutaneously in the anterior aspect of the thigh using a 27 French gauge needle for 5 days, while for infants in control group an equivalent amount of normal saline solution was injected as the placebo. All the necessary clinical and para-clinical diagnostic and therapeutic proceedings were under the supervision of physicians.

During the study period, infants were monitored for their respiratory status (required oxygen and required assisted ventilation such as nasal Continuous Positive Airway Pressure or mechanical ventilation), feeding tolerance (ml/Kg), abdominal distention and incidence of adverse reactions such as fever, restlessness, thrombocytopenia, and oxygen dependency. To evaluate the prevalence of documented sepsis, on the fourteenth day of study, blood culture and urine or spinal fluid specimens cultures (in necessary cases) of infants in both groups were obtained and also the information of final status of infants including mortality, Necrotizing Entrocolitis (NEC) , feeding tolerance, weight gain and respiratory status was recorded in the questionnaire. Laboratory assistants or other assessors of the results in this study did not know which participants are subject to which procedure.

**Statistical Analysis**

The data were analyzed using SPSS statistical software (version 15). Chi-square test or Fisher exact test was used for data analysis of qualitative variables and mean values were compared using independent t-test. Differences were considered significant at P-value <0.05.

## Results

Among the 46 infants (Figure 1), 26 (56%) were males, and 20 (44%) were female which is not statistically significant. There were no significant difference between treated and control infants in birth weight, gestational age, apgar score at the first and fifth minute and ANC at the beginning of study (Table I).The mean gestational age in GCSF and control group were 33±2and 32±2 weeks ,respectively. Mean of ANC in GCSF and control group were 2014 and 2595 per micro liter, respectively.

In the GCSF group, one death was reported at 7^th^ day of study, however, no mortality was observed in the control group (P-value = 1.00). None of the blood cultures were positive in both on the fourteenth day. 

The mean weight gain for GCSF group was 172 gr and 205 gr in the control group on the fourteenth day, which was not statistically significant (P-value = 0.982).The mean of hospital stay in GCSF group was 25 (SD = 6) days and in control group was 29 (SD = 7) days, and this difference was statistically significant (P-value = 0.042) (Table II).

No possible drug side effects were observed on daily checks. Only one case with WBC of30800/mm³was reported in GCSF group in the second week of study. The mean ANC in GCSF group was 4,062/μl (SD = 2498) and 3,034/μl (SD = 1150) in control group, which the difference between two groups was not statistically significant (P-value = 0.067).However, the change in ANC on fourteenth day after inclusion in the study, Compared to the first day of enrollment, was significant for both groups (P-value < 0.05). Only one case in treatment group and three cases in control group needed respiratory support which showed feeding intolerance, but the difference was not significant (P-value = 0.608).

**Table I:** baseline characteristics of study subjects

**Variable**	**GCSF group (n=23)**	** Control group ( n=23) **	**P-value**
**Male n( % )**	12 ( 52 .2 % )	14 ( 60 .9 % )	0.383
**BW( gr )** **mean±SD**	1540 ± 382	1408 ± 340	0.214
**GA( Weeks ) mean±SD**	33 ± 2	32 ± 2	0.233
**Apgar score** **1** ^st^ ** min** **mean±SD**	7 ± 2	8 ± 1	0.251
**Apgar score** **5** ^th^ ** min** **mean±SD**	9 ± 1	9 ± 1	0.656
**ANC of beginning of study (Neut/μL)** **mean±SD**	2014 ± 928	2595 ± 1025	0.602

## Discussion

Based on the results of the present study, the addition of GCSF to conventional treatment schedule of premature infants with neutropenia and clinical sepsis could shorten the length of hospitalization.

Despite advances in neonatal intensive care and advancement in the production of broad-spectrum antibiotics, sepsis is still an important cause of mortality and morbidity in newborns, especially in premature infants, so reducing the mortality of sepsis is a great interest for researchers. According to findings, neutropenia was transient in neonates, and the mean duration of neutropenia without treatment was reported to be about 36 hours (16), but this time might be sufficient for occurrence of sepsis. So, several studies have been conducted to assess the effects of GCSF. The use of GCSF first reported in 1991 to a 654 gram weight premature infant with multiple episodes of sepsis and absolute neutrophil counts of less than1,000 cells/μL. The infant was treated subcutaneously with10 μg/kg per day of GCSF, so the ANC kept between 8,000 to 12,000. Finally, the infant discharged from hospital with good 

general condition and ANC higher than 2,000 cells /μL (17). Afterward researchers in several studies have evaluated the molecular and experimental effects of GCSF, and confirmed that this factor could improve the quality and quantity of neonatal neutrophils (20, 21, and 22). Subsequently, researchers have design studies to evaluate the clinical effect of GCSF in prognosis or reduction in mortality of premature infants.

Although long term antibiotic-therapy, due to some limitations such as maintaining sterile conditions particularly in preterm infants with neutropenia is criticalin developing countries. But, at the present study ,high concentrations of antibiotics in the culture medium is considered as a negative factor for the growth of micro-organisms (23) and so a confounding factor in the assessment of sepsis by blood culture in the present study. Therefore due to the different therapeutic managements of neonatal sepsis and administration of antibiotics in developing countries, it is recommended to assess the results of blood culture inattention to the other clinical and Para- clinical results.

In a similar study conducted by Gathwala and colleagues in India, GCSF treatment, could significantly reduce all-cause mortality rate (21). In another study conducted in London, Russel et al studied 28 neonates with birth weight of 500-1500 grams, ANC ≤5000/mm³ and clinical evidence of sepsis, the number of deaths was significantly fewer in the group receiving GCSF (15). In another study in Brazil on44 preterm neonates with sepsis, who were <5 days old and weighing 500-2000 gr, the rate of mortality was not significantly different between the two groups, but the occurrence of a subsequent nosocomial infection was significantly reduced in the treatment group (22). Since the results are conflicting, some researchers have deduced that perhaps this drug is effective in certain groups (Small for Gestational Age infants, BW less than 1500 or GA less than 32 weeks) (24). Therefore, additional well-designed trials are needed to confirm these early results.

## Conclusion

While the effects of GCSF on blood indexes and neutrophils function is of agreement, the results of this study showed that a 5 day period of GCSF therapy in premature infants with neutropenia that present with clinical sepsis is safe and can reduce the length of hospitalization. In other studies it is needed to be designed with different methods to confirm its therapeutic effect.
